# Expression Patterns, Genomic Conservation and Input Into Developmental Regulation of the GGDEF/EAL/HD-GYP Domain Proteins in *Streptomyces*

**DOI:** 10.3389/fmicb.2018.02524

**Published:** 2018-10-23

**Authors:** Mahmoud M. Al-Bassam, Julian Haist, Sara Alina Neumann, Sandra Lindenberg, Natalia Tschowri

**Affiliations:** ^1^Division of Host-Microbe Systems and Therapeutics, Department of Pediatrics, University of California, San Diego, San Diego, CA, United States; ^2^Institut für Biologie/Mikrobiologie, Humboldt-Universität zu Berlin, Berlin, Germany

**Keywords:** c-di-GMP, development, *Streptomyces*, GGDEF, EAL, HD-GYP

## Abstract

To proliferate, antibiotic-producing *Streptomyces* undergo a complex developmental transition from vegetative growth to the production of aerial hyphae and spores. This morphological switch is controlled by the signaling molecule cyclic bis-(3′,5′) di-guanosine-mono-phosphate (c-di-GMP) that binds to the master developmental regulator, BldD, leading to repression of key sporulation genes during vegetative growth. However, a systematical analysis of all the GGDEF/EAL/HD-GYP proteins that control c-di-GMP levels in *Streptomyces* is still lacking. Here, we have FLAG-tagged all 10 c-di-GMP turnover proteins in *Streptomyces venezuelae* and characterized their expression patterns throughout the life cycle, revealing that the diguanylate cyclase (DGC) CdgB and the phosphodiesterase (PDE) RmdB are the most abundant GGDEF/EAL proteins. Moreover, we have deleted all the genes coding for c-di-GMP turnover enzymes individually and analyzed morphogenesis of the mutants in macrocolonies. We show that the composite GGDEF-EAL protein CdgC is an active DGC and that deletion of the DGCs *cdgB* and *cdgC* enhance sporulation whereas deletion of the PDEs *rmdA* and *rmdB* delay development in *S. venezuelae*. By comparing the pan genome of 93 fully sequenced *Streptomyces* species we show that the DGCs CdgA, CdgB, and CdgC, and the PDE RmdB represent the most conserved c-di-GMP-signaling proteins in the genus *Streptomyces*.

## Introduction

*Streptomyces* are high G + C Gram-positive Actinobacteria with a great interest for medicine and industry, representing the most abundant natural source of antibiotics and other medically relevant secondary metabolites. Genome mining has revealed that they contain a huge number of natural product gene clusters that are silent under laboratory conditions and therefore represent a promising reservoir for the discovery of novel bioactive compounds (Rigali et al., 2018). *Streptomyces* mostly inhabit terrestrial ecosystems where they play an important role for nutrient recycling by secretion of different hydrolytic enzymes such as chitinases and cellulases (Ventura et al., 2007). During the vegetative phase, *Streptomyces* grow by extension of their hyphal tips and by initiating new lateral branches. It is due to this mode of growth that vegetative-phase colonies consist of tightly interwoven hyphae leading to a compact mycelium that appears bald and shiny on solid medium. As the colony matures, reproductive aerial hyphae are formed on the surface, which differentiate into long chains of pre-spore compartments and result in a fuzzy and white appearance of the colony. At the end of the life cycle, these compartments mature into pigmented spores that give *Streptomyces* colonies a characteristic color. Spores disperse, thus allowing the organism to spread to new environments. The correct order of morphological and physiological differentiation events is controlled by a fine-tuned cascade of Bld and Whi regulators (Bush et al., 2015). Bld regulators control the switch from the vegetative to aerial hyphae and Whi regulators are required for the transition from aerial hyphal growth to sporulation (Flärdh and Buttner, 2009; McCormick and Flärdh, 2012).

Recently, it has been discovered that some *Streptomyces* can adapt other modes of growth that deviate from the typical developmental cycle. When co-cultured with yeast, many *Streptomyces* spp. show exploratory growth by forming non-branching vegetative hyphae that lead to rapid colony expansion over solid surfaces (Jones et al., 2017). These explorer cells do not form aerial mycelium or spores and the exploratory behavior does not depend on known Bld or Whi regulators. In contrast, some *Streptomyces venezuelae* mutants undergo precocious hypersporulation, which bypasses the formation of aerial hyphae and leads to colonies that consist only of spores. These strains include *bldD* and *bldO* mutants and a strain that overexpresses a c-di-GMP phosphodiesterase (PDE) (Tschowri et al., 2014; Bush et al., 2017).

The developmental master regulator BldD is a two-domain DNA-binding repressor protein that is mainly present in the vegetative growth phase and directly controls the expression of 167 target genes in *S. coelicolor* (den Hengst et al., 2010). The BldD-regulon encompasses other *bld* regulators, several *whi* regulators, and genes encoding components of the cell division and chromosome segregation machineries. The critical physiological function of BldD is to shut off sporulation genes during vegetative growth but to allow expression of these genes in the later developmental stages for sporulation. BldD can only bind to the target DNA when it is in complex with the signaling molecule c-di-GMP. A c-di-GMP tetramer binds to the C-terminal domain of BldD, leading to the formation of a stable BldD dimer and an active repressor (Tschowri et al., 2014; Tschowri, 2016; Schumacher et al., 2017).

The ubiquitous bacterial second messenger c-di-GMP is metabolized by three different classes of proteins with specific domains that are named after conserved amino acids in their active sites (A-site). Formation of c-di-GMP from two molecules of GTP is mediated by GGDEF domain-containing diguanylate cyclases (DGCs). In close proximity to the A-site, most active DGCs also carry a conserved RxxD motif, termed the inhibitory site (I-site), which is involved in feedback control of DGC activity through product inhibition (Chan et al., 2004; Paul et al., 2007). Specific PDEs, which harbor the EAL domain or the less frequent HD-GYP domain, degrade the cyclic dinucleotide molecule (Christen et al., 2005; Schmidt et al., 2005; Ryan et al., 2006). Often, domains with opposite enzymatic activities are found in the same protein as GGDEF-EAL or GGDEF-HD-GYP tandems. Typically, in such proteins one of the two domains is enzymatically inactive, but a few bifunctional DGCs/PDEs exist (Tarutina et al., 2006). Often, these proteins also have diverse sensory domains (e.g., PAS, GAF) that enable cells to adjust second messenger levels in response to different environmental cues (Römling et al., 2013). Recent studies demonstrated that in *S. coelicolor* CdgA and CdgB are functional DGCs and RmdA and RmdB have PDE activity. Overexpression of either CdgA or CdgB and the deletion of *cdgB* inhibit morphological and physiological differentiation in *S. coelicolor* (den Hengst et al., 2010; Tran et al., 2011). Single deletions of either *rmdA* or *rmdB* delay *S. coelicolor* development, which is completely blocked in the *rmdA rmdB* double mutant (Hull et al., 2012).

A global analysis of an entire set of c-di-GMP metabolizing proteins in any *Streptomyces* strain is still lacking. Here, we have used *S. venezuelae* as a model species for studies of *Streptomyces* morphological development, which sporulates in liquid culture as opposed to the traditional model species *S. coelicolor*. As such, we aimed to determine which of the 10 c-di-GMP turnover enzymes affect development in these bacteria. To address this question, we have systematically deleted each of the 10 GGDEF, EAL, and HD-GYP proteins encoding genes and show that the DGCs CdgB and CdgC and the PDEs RmdA and RmdB are the only c-di-GMP-signaling enzymes that affect differentiation under tested conditions. By comparing the conservation of c-di-GMP metabolism genes in the genomes of 93 fully sequenced *Streptomyces* strains, we find that CdgB, CdgC, and RmdB belong to the most widely distributed proteins and thus very likely have a conserved role in the genus *Streptomyces*.

## Results

### C-di-GMP Turnover Proteins in *S. venezuelae*

*Streptomyces venezuelae* has 10 chromosomally encoded and one plasmid-encoded protein involved in c-di-GMP metabolism (Table 1). Four genes encode proteins with a GGDEF domain only, five genes encode composite proteins containing a GGDEF domain and an EAL domain, and two genes encode for proteins with a HD-GYP domain. For the genes encoding the GGDEF and EAL domain proteins we decided to keep the “*cdg*” designation, which stands for “c-di-GMP”, as is the case for the *cdgA* and *cdgB* homologues in *S. coelicolor*. We suggest *hdgA* and *hdgB* (HD-GYP), respectively, for *vnz24090*, coding for a conserved HD-GYP domain protein, and *vnz24095* encoding a degenerate HD-GYP domain protein missing the key GYP residues.

**Table 1:** Domain architecture and designations for GGDEF/EAL/HD-GYP domain-encoding genes in *Streptomyces venezuelae*.

Gene Name	*vnz* Number (StrepDB)	*sven* Number (StrepDB)	Domain Architecture	Enzymatic Activity	References/Comments
*cdgA*	*vnz12760*	*sven2604*	PAS-PAC-GGDEF-EAL	DGC	(den Hengst et al., 2010)
*cdgB*	*vnz19920*	*sven4034*	GAF-PAS-PAC-GGDEF	DGC	(Tran et al., 2011)
*cdgC*	*vnz25635*	*sven5187*	10TM-PAS-PAC-GGDEF-degEAL	DGC	This study
*cdgD*	*vnz19760*	*sven3999*	GGDEF	^∗^DGC	
*cdgE*	*vnz22740*	*sven4602*	GAF-GGDEF	^∗^DGC	
*cdgF*	*vnz02140*	*sven0451*	10TM-PAS-PAC-GGDEF-EAL	^∗^DGC/PDE	
*rmdA*	*vnz33675*	*sven6830*	PAS-PAC-GGDEF-EAL	PDE	(Hull et al., 2012)
*rmdB*	*vnz25525*	*sven5165*	6TM-GGDEF-EAL	PDE	(Hull et al., 2012)
*hdgA*	*vnz24090*	*sven4872*	6TM-HD-GYP	^∗^PDE	
*hdgB*	*vnz24095*	*sven4873*	6TM-degHD-GYP	^∗^none	
*pcdgG*	*pvnz37430*		GGDEF	^∗^DGC	on plasmid

*^∗^Predicted activity. deg, degenerate; TM, transmembrane.*

The DGC function of CdgA and CdgB and the PDE activity of RmdA and RmdB of *S. coelicolor* have been demonstrated experimentally (den Hengst et al., 2010; Tran et al., 2011; Hull et al., 2012). CdgC has a highly degenerate EAL domain but a conserved GGDEF domain. CdgD, CdgE, and the plasmid-encoded pCdgG contain conserved GGDEF domains and can be assigned DGC function. The composite protein CdgF may represent a bifunctional protein since it has all the key residues in both the GGDEF and EAL domains to be enzymatically active. CdgC, CdgF, and RmdB are predicted to contain transmembrane helices. CdgA, CdgB, CdgC, CdgE, CdgF, and RmdA, all contain either PAS, PAC, GAF, or a combination of these N-terminal signaling domains but their specific functions and the signals they perceive remain unknown.

### Expression Patterns of GGDEF-, EAL-, HD-GYP-Domain Proteins in *S. venezuelae*

**FIGURE 1:**
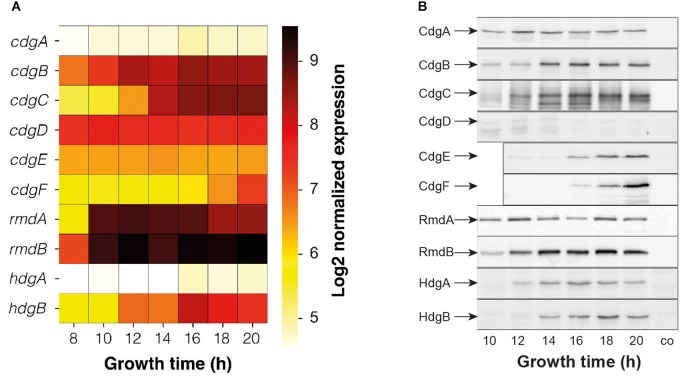
Expression profiles of c-di-GMP metabolism genes and proteins during submerged sporulation of *S. venezuelae.* **(A)** Expression data were extracted from microarray analyses in Bibb et al. (2012) and normalized with quantile normalization and median polish using the RMA method. Expression values are shown as Log2. **(B)** Western blot analysis of FLAG-tagged proteins. GGDEF/EAL/HD-GYP-domain proteins were FLAG-tagged and expressed in the corresponding mutants. Protein samples were harvested after indicated time of growth and following amounts of total protein were used for Western blotting: 5 μg for CdgB-FLAG; 10 μg for CdgE-FLAG, RmdB-FLAG and HdgB-FLAG; 15 μg for CdgA-FLAG, CdgC-FLAG, and RmdA-FLAG; 20 μg for CdgD-FLAG, CdgF-FLAG, HdgA-FLAG. co, wildtype sample as control.

When grown in liquid, *S. venezuelae* begins to differentiate after ca. 14 h of growth when vegetative hyphae start to fragment and spores begin to form. To determine the stages of growth that individual GGDEF-, EAL-, HD-GYP proteins are present and thus may affect development, we have analyzed their expression patterns at both the transcript and protein levels (Figure 1). Time-course microarray experiments were performed in the study by Bibb et al. (2012), to determine the transcriptional profile of *S. venezuelae* during submerged sporulation. Seven samples were taken at 8, 10, 12, 14, 16, 18, and 20 h of growth, spanning the complete morphological differentiation states of the bacterium (Bibb et al., 2012). We have extracted the transcription data for the chromosomally encoded c-di-GMP signaling genes from this microarray experiment and carried out quantile normalization and median polishing using the RMA method. The expression profiles show that *rmdA* and *rmdB*, coding for active PDEs, represent the most highly expressed genes in the analyzed set. Their transcripts were abundant throughout the life cycle from vegetative growth to sporulation. Similarly, *cdgB* and *cdgD*, coding for an active and a predicted DGC, respectively, were also expressed in all developmental stages. Transcripts of *cdgC* accumulated along the life cycle and achieved highest levels in the sporulation phase, and this is also true for *hdgB*, coding for a degenerate HD-GYP protein. *cdgA*, *cdgE*, *cdgF*, and *hdgA* were the least expressed genes under the conditions tested.

To examine the expression of all the c-di-GMP-metabolizing proteins, we constructed alleles expressing C-terminally FLAG-tagged protein variants under the control of their native promoters. These alleles were cloned into the integrative vector pIJ10770 (Schlimpert et al., 2017), introduced into the corresponding deletion mutants at the ΦBT1 attachment site, and analyzed by Western blotting using a monoclonal anti-FLAG antibody. In each case, about 300 nucleotides upstream of the coding region were included in the constructs, in an attempt to incorporate the entire regulatory region. However, genomic organization suggests that *cdgD* is very likely arranged in one operon with *vnz19765*; therefore, the *cdgD*-FLAG construct contains the entire region upstream of *cdgD* up to *vnz19770*. Similarly, *rmdA* appears to be in an operon with *vnz33670* and *hdgB* is very likely in an operon with *hdgA* and therefore these genes plus ca. 300 bp of their upstream region were included in the *rmdA*-FLAG and *hdgB*-FLAG constructs.

Due to pronounced differences in expression levels, the total protein amount used in this analysis was adjusted for individual proteins. For most of the GGDEF/EAL/HD-GYP domain proteins, the FLAG-tagged protein levels show that – considering the total protein loaded – the DGC CdgB and the PDE RmdB represent the most abundant c-di-GMP-signaling enzymes and CdgD, CdgF, and HdgA the least abundant c-di-GMP metabolizing proteins in *S. venezuelae* (Figure 1B). The protein levels of CdgA and RmdA appear to be constant in all developmental stages, whereas other proteins accumulate along the life cycle. Despite high transcription of *cdgD* (Figure 1A), we could hardly detect CdgD-FLAG in our Western blot analysis (Figure 1B). This suggests either that we have not included a relevant regulatory region in our construct or some post-transcriptional mechanism negatively affects CdgD levels.

CdgC, CdgF, RmdB, HdgA, and HdgB are predicted to contain transmembrane helices and thus to be bound to the cell membrane (Table 1). To experimentally validate this prediction, we separated the whole cell lysate from relevant strains expressing the FLAG-tagged alleles into soluble and membrane fraction using ultracentrifugation. Using the anti-FLAG antibody and Western blotting, we detected CdgC-FLAG, CdgF-FLAG, RmdB-FLAG, HdgA-FLAG, and HdgB-FLAG only in the membrane fraction (Supplementary Figure S2), confirming their membrane-bound localization.

### CdgB, CdgC, RmdA, and RmdB Impact Morphological Differentiation in *S. venezuelae*

**FIGURE 2:**
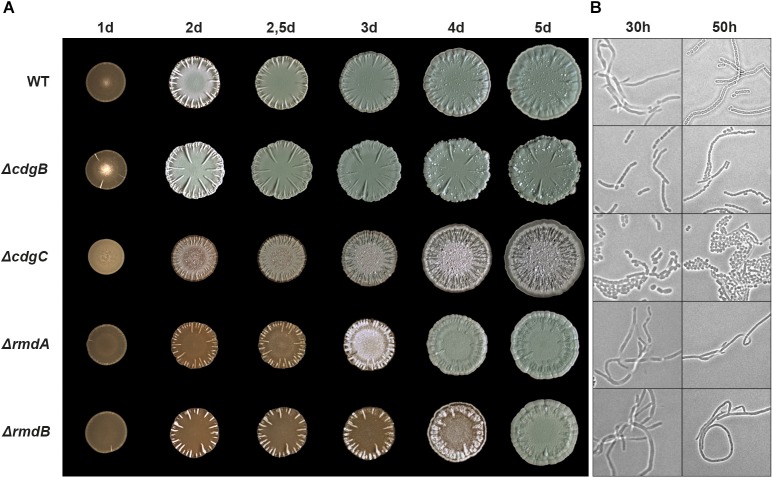
Morphological differentiation is accelerated by the deletion of *cdgB* and *cdgC* and delayed by the deletion of *rmdA* and *rmdB*. **(A)** Time-course development of *S. venezuelae* wildtype and mutant macrocolonies grown on MYM agar for 5 days at 30°C. **(B)** Phase contrast microscopy images showing coverslip imprints from the upper layer of the macrocolonies grown for 30 h and 50 h in (A).

To determine which of the DGC(s) and PDE(s) are involved in the regulation of differentiation in *S. venezuelae*, we created deletion mutants for all 10 chromosomal genes encoding GGDEF/EAL/HD-GYP proteins. Macrocolony morphology analysis of mutant strains grown for up to 5 days on MYM agar at 30°C revealed that deletions of *cdgB*, *cdgC*, *rmdA*, and *rmdB* result in morphological phenotypes which deviate from the wildtype phenotype (Figure 2 and Supplementary Figure S3). Complementation of these phenotypes with the FLAG-tagged alleles shows that the FLAG-tagged proteins are functional, and that the mutations are congenic with the wildtype strain (Supplementary Figure S4). *S. venezuelae* colonies turn green upon spore maturation due to the synthesis of a spore specific polyketide pigment. As shown in Figure 2, the *cdgB* mutant became green and produced mature spores reproducibly faster than the wildtype. Coverslip impression taken after 30 h of growth showed that Δ*cdgB* had already produced spores organized in characteristic chains whereas the wildtype strain still mainly consisted of non-divided filaments. Furthermore, the *cdgB* mutant formed colonies that are thicker compared to the wildtype colony and produced elaborated radial ridges on the surface.

Deletion of *cdgC* resulted in the most striking macrocolony phenotype. This strain formed very flat colonies that did not turn green even after prolonged incubation and formed many radial wrinkle-like structures on the colony surface. The *cdgC* mutant did not produce the classical hair-like aerial hyphae but sporulated precociously, as shown by coverslip images taken after 30 h of incubation (Figure 2). However, the spores were not in chains but appeared as an irregular mass, reminiscent of the phenotype of an *S. venezuelae* strain that overproduces the active *Escherichia coli* PDE PdeH (YhjH), leading to hypersporulation (Tschowri et al., 2014). Similarly, *bldD* and *bldO* deletion mutants bypass aerial mycelium formation and form spores precociously (Tschowri et al., 2014; Bush et al., 2017). The pleiotropic phenotype of the *cdgC* mutant suggests that CdgC acts as one of the major determinants of c-di-GMP levels in the cell.

Deletion of either *rmdA* or *rmdB* severely inhibited *S. venezuelae* development, resulting in a prolonged bald morphology. The *rmdA* and *rmdB* mutant macrocolonies matured after 4 and 5 days, respectively, whereas the wildtype strain formed mature spores after just 2.5 days (Figure 2A). As expected, coverslip impression images show that, after 50 h of incubation, the *rmdA* and *rmdB* colonies consisted of pure vegetative hyphae (Figure 2B). Interestingly, the *rmdB* mutant needed 1 day longer than the *rmdA* mutant to become green, i.e., develop mature spores. The RmdA and RmdB proteins share conserved GGDEF and EAL domains but their N-terminal domains differ, with RmdA having a PAS-PAC domain and RmdB having a transmembrane domain (Table 1). The phenotypic similarity of the two mutants suggests that the functions of these two genes are not redundant, which is also consistent with their spatial segregation since RmdA is a cytosolic and RmdB a membrane-associated protein (Supplementary Figure S2).

### CdgC Is an Active DGC *in vitro* and the GGDEF Active Site Is Essential for Its *in vivo* Function

**FIGURE 3:**
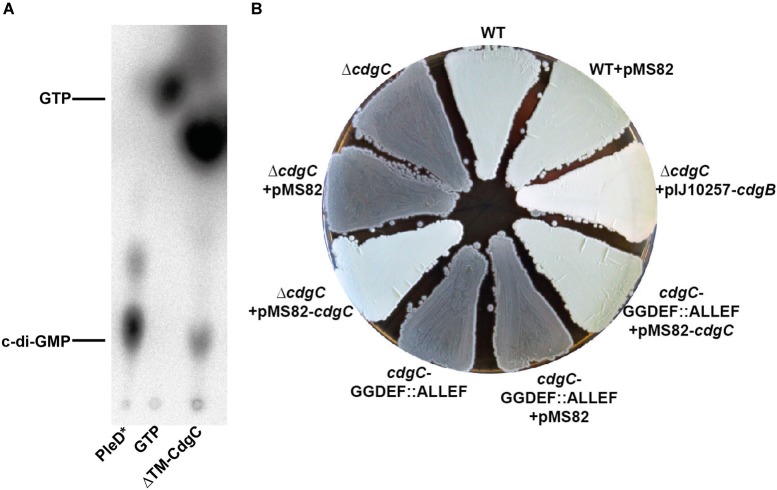
CdgC is an active DGC. **(A)** Synthesis of c-di-GMP from GTP was assayed *in vitro* using purified cytosolic fraction of CdgC (ΔTM-CdgC; TM: transmembrane) and PleD^∗^ and visualized by thin layer chromatography. **(B)** The *cdgC* mutant and a strain carrying a chromosomal *cdgC* version with the ALLEF motif in the active site show green wildtype morphology when complemented with *cdgC* from the pMS82 vector but remain gray and do not form aerial mycelium with the empty vector. Overexpression of CdgB can partially suppress the *cdgC* deletion. Strains were grown for 3 days on MYM agar.

The architecture of CdgC strongly suggests that this protein is an active DGC since it contains a highly conserved GGDEF domain but a degenerate EAL domain missing all the key residues involved in c-di-GMP cleavage. However, the enzymatic activity of CdgC has not yet been addressed in any study. To validate the predicted DGC activity of CdgC experimentally, we have purified the N-terminally His6-tagged cytosolic fraction of CdgC (ΔTM-CdgC) and tested it for *in vitro* DGC activity. A mutationally activated form of the *C. crescentus* PleD protein (PleD^∗^) served as our positive control (Paul et al., 2007). We used [^32^P]-GTP as substrate and separated the reactions by thin layer chromatography (TLC) (Christen et al., 2005). As shown in Figure 3A, ΔTM-CdgC produced c-di-GMP from GTP and is thus an active DGC.

We confirmed DGC activity of CdgC also genetically. For that, we mutagenized the GGDEF active site to ALLEF in the chromosomal locus of *cdgC* and found that the resulting strain was phenotypically indistinguishable from the strain containing the complete *cdgC* deletion (Figure 3B). As in the null mutant, *S. venezuelae* carrying the mutagenized *cdgC* allele did not form aerial mycelium, remained gray on MYM plates even after prolonged (5 days) incubation (Figure 3B and data not shown) and showed precocious sporulation. For complementation, we cloned *cdgC* under the control of its native promoter into the integrative pMS82 vector and introduced this construct by conjugation into the Δ*cdgC* mutant and *S. venezuelae* expressing CdgC with the ALLEF motif instead of GGDEF in the active site. In contrast to the empty vector pMS82, expression of *cdgC* from the ΦBT1 phage integration site fully complemented both mutants.

To analyze whether the developmental phenotype caused by *cdgC* deletion can be suppressed by a heterologous DGC, we overexpressed CdgB from *S. coelicolor* using pIJ10350 described in Tran et al. (2011). pIJ10350 is a derivative of the integrative pIJ10257 vector containing *cdgB* under control of the strong constitutive *ermEp*^∗^ promoter (Tran et al., 2011). Our data, presented in Figure 3B show that the *cdgC* phenotype could be partially suppressed by overexpressing the *S. coelicolor* CdgB, supporting the conclusion that changes in c-di-GMP levels is the main reason behind the hypersporulation phenotype of the *cdgC* mutant. However, the *cdgC* mutant overexpressing CdgB remains white even after 5 days of incubation. This could be due to differences in c-di-GMP levels resulting from *cdgB* overexpression, or to a CdgC-specific contribution to the developmental program that cannot be complemented by c-di-GMP synthesis derived from the overexpression of a heterologous DGC. Altogether, these results show that CdgC is an active DGC *in vitro* and fulfills its biological function via the GGDEF active site, but that it may also have a secondary role that is specific to this protein.

### Distribution of the *S. venezuelae* GGDEF/EAL/HD-GYP Orthologs in the *Streptomyces* Pan Genome

**FIGURE 4:**
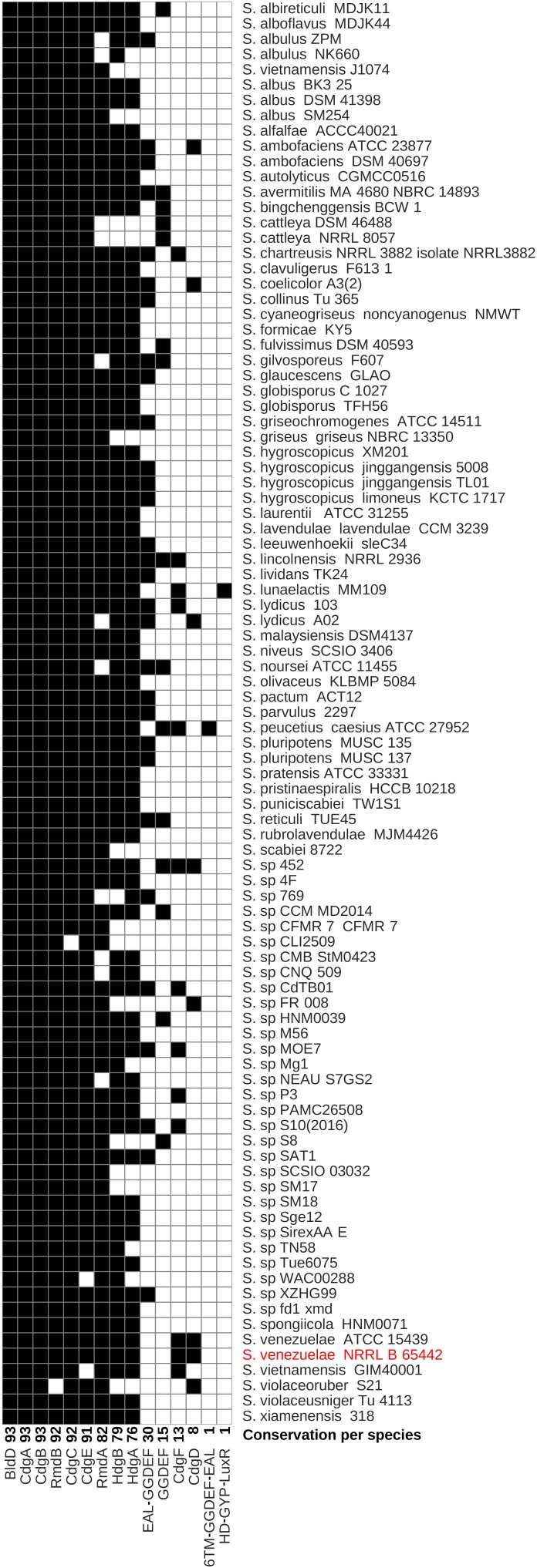
Distribution of c-di-GMP turnover genes in *Streptomyces spp*. Species are sorted in alphabetical order and the model streptomycete, *S. venezuelae*, is highlighted in red. The conservation of each gene across *Streptomyces* spp. is shown next to each gene name. Black boxes in each column represent the presence of gene orthologs calculated using the BPGA pipeline or blastp. White boxes represent absence of orthologous genes. TM, transmembrane.

Having learned that CdgB, CdgC, RmdA, and RmdB have crucial roles in controlling the coordinated progress of the *S. venezuelae* life cycle, we wondered to what extent these four proteins are conserved across *Streptomyces* species. To identify the core set of c-di-GMP turnover proteins and their overall distribution in the genus *Streptomyces*, we analyzed the pan genome of 93 *Streptomyces* species with completely sequenced genomes available at the NCBI database^1^. The genomes used in this study, and their accession numbers are listed in Supplementary Table S2. We functionally annotated the resulting gene clusters using the COG database and then searched for the relevant COG annotations for GGDEF, EAL, and HD-GYP domains (see Materials and Methods). We also included the developmental master regulator BldD in the analysis as it directly binds c-di-GMP and thus represents a key c-di-GMP signaling component in *Streptomyces*. We set chromosomally encoded c-di-GMP metabolism proteins of our model organism *S. venezuelae* as reference and used 45% identity cutoff to designate ortholog clusters (Chaudhari et al., 2016). By using these parameters, we identified 15 different GGDEF/EAL/HD-GYP domain proteins in the *Streptomyces* pan genome (Figure 4 and Supplementary Table S3). The number of c-di-GMP-turnover proteins per strain varies from 5 (e.g., in *S. sp* CLI2509) up to 12 (e.g., in *S. lincolnensis* NRRL2936) with most strains having nine GGDEF/EAL/HD-GYP domain proteins.

*bldD*, *cdgA*, and *cdgB* are present in all 93 *Streptomyces* species (Figure 4), and we defined these as “core” genes. The genes encoding the PDE RmdB, the DGC CdgC, and the putative DGC CdgE are present in at least 91 of the analyzed strains and belong to the “soft-core” category of genes (see Materials and Methods; Figure 4 and Supplementary Table S3). RmdB is only absent in *S. violaceoruber* S21 and CdgC cannot be found in *Streptomyces sp* CLI2509. *S. vietnamensis* GIM40001 and *Streptomyces sp* WAC00288 lack a CdgE ortholog. The PDE RmdA is present in 82 fully sequenced *Streptomyces* and coexists with RmdB in 81 out of 93 species. However, *S. violaceoruber* S21, lacking RmdB, contains the PDE RmdA but no other EAL domain proteins, suggesting that RmdA is the major EAL-type PDE in this strain. We found HdgA orthologs in 76 and HdgB orthologs in 79 strains, respectively. Thirteen strains do not have any of the HD-GYP proteins, supporting the hypothesis that HD-GYP proteins do not fulfill a conserved function in c-di-GMP metabolism in *Streptomyces*.

Since we have learned that *cdgB*, *cdgC*, *rmdA*, and *rmdB* are c-di-GMP-signaling genes that affect development in *S. venezuelae* and are widely distributed in the genus *Streptomyces*, we wondered whether these genes are encoded in a conserved genetic context. As such, we calculated the bidirectional blast hit of the three genes located both downstream and upstream of *bldD*, *cdgB*, *cdgC*, *rmdA*, and *rmdB* and plotted the blast identity percent as heatmaps. Our data show that, apart from *rmdA*, the analyzed genetic regions are highly conserved (Supplementary Figure S5). The overall conservation of the genetic context of these c-di-GMP metabolism genes further supports the accuracy of our orthologue groups.

### Extended Repertoire of GGDEF/EAL/HD-GYP Genes in the *Streptomyces* Pan Genome

#### EAL-GGDEF Domain Protein

Thirty of the analyzed *Streptomyces* strains encode for a c-di-GMP signaling protein that cannot be found in the *S. venezuelae* reference genome but is homologous to e.g., SAZ_08970 protein from *S. albulus* ZPM (Figure 4 and Supplementary Table S3). The domain architecture of this protein is unique in *Streptomyces*. It contains an EAL domain on the N-terminus followed by a GGDEF domain in the C-terminal region. In the EAL domain, one key glutamic acid essential for the formation of the PDE active center is missing and the GGDEF motif is replaced by GGDDF (data not shown).

#### GGDEF Domain Proteins

Fifteen *Streptomyces* strains have stand-alone cytoplasmic GGDEF-domain proteins that are structurally similar to *S. venezuelae* CdgD but do not pass 45% sequence identity cut-off. All except TUE45_01367 from *S. reticuli* TUE45 have a conserved GGDEF motif and can be assigned DGC function. Notably, 13 of these strains do not have a CdgD protein, suggesting that these single GGDEF domains might be evolved variants of CdgD. However, *Streptomyces* sp. 452 contains two such proteins: DC008_23740, which is 61% identical to CdgD, and DC008_23810, which is 44% identical to CdgD, suggesting that the *DC008_23810* arose by gene duplication.

#### 6TM-GGDEF-EAL Domain Protein

In addition to the c-di-GMP turnover proteins present in *S. venezuelae*, *S. peucetius* subsp caesius ATCC27952 also has a GGDEF-EAL domain protein (CGZ69_35130) that is not present in *S. venezuelae* or any other sequenced *Streptomyces* strain. This protein is to 43% identical to *S. venezuelae* CdgF and is therefore not considered as a CdgF ortholog. However, as in CdgF, the GGDEF and EAL domains are highly conserved, suggesting that this protein might be bifunctional as well. Moreover, CGZ69_35130 is also predicted to be membrane-bound but seems to have six transmembrane helices instead of the 10 predicted for CdgF. Taken together, it is very likely that this gene arose by gene duplication and represents a modified copy of *cdgF*.

#### HD-GYP-LuxR Domain Protein

*Streptomyces lunaelactis* MM109 has acquired a c-di-GMP metabolism protein with a unique domain structure. SLUN_31535 has an N-terminal HD-GYP domain and a C-terminal HTH_LuxR-like DNA binding domain. This protein is 32% identical to HdgA from *S. venezuelae* but does not have the key GYP residues for c-di-GMP PDE activity.

## Discussion

In the genus *Streptomyces*, the conserved master regulator BldD acts as a central hub that integrates c-di-GMP signals into the developmental program. During vegetative growth, it binds tetrameric c-di-GMP, dimerizes, and shuts off sporulation genes (Tschowri et al., 2014). *S. venezuelae* has 10 chromosomally encoded GGDEF/EAL/HD-GYP proteins. Except for HdgB, which has a degenerate active site in the HD-GYP domain, all the other proteins have conserved c-di-GMP-metabolizing domains and the potential to control c-di-GMP sensed by BldD or by a putative yet undiscovered additional c-di-GMP effector with a role in *Streptomyces* development. However, here we show that in *S. venezuelae* only the DGCs CdgB and CdgC and the PDEs RmdA and RmdB have a significant impact on morphological differentiation. The conservation of CdgB, CdgC, and RmdB reflects their importance in controlling *Streptomyces* sporulation, and we conclude that orthologs of these genes are likely to play equivalent roles across all *Streptomyces* species.

Deletion of the DGC genes *cdgB* and *cdgC* leads to enhanced sporulation, suggesting that BldD is less active in these mutants, likely due to reduced c-di-GMP levels. However, we see striking differences in the extent and mode of sporulation in these two mutants. Despite producing spores earlier than the wildtype, the *cdgB* mutant still undergoes the full life cycle including the erection of aerial hyphae that ultimately turn into spore chains (Figure 2). In contrast, the *cdgC* mutant bypasses aerial mycelium formation and forms spores directly from the hyphae on the surface of a *S. venezuelae* colony. Inactivation of *cdgC* strikingly mimics the hypersporulation phenotype seen when *bldD* is deleted or the PDE PdeH is overexpressed (Tschowri et al., 2014), suggesting that BldD becomes inactive as a repressor in the *cdgC* mutant because c-di-GMP levels are too low to drive dimerization of BldD. If so, CdgB and CdgC seem to influence c-di-GMP levels to different degrees, with CdgC being the most active DGC during vegetative growth. Alternatively, CdgC and BldD may form a locally acting module as reported for other c-di-GMP-dependent systems (Lindenberg et al., 2013; Sarenko et al., 2017). However, using bacterial two-hybrid analysis, we could not detect any interaction between CdgC and BldD (data not shown), and the observation that the *cdgC* phenotype can, at least partially, be suppressed by expressing CdgB, is also inconsistent with a locally acting module. CdgC is membrane bound (Supplementary Figure S2) and therefore could possibly receive activity-modulating signals via the N-terminal transmembrane or PAS-PAC sensory domains. Moreover, the degenerate EAL domain may affect the GGDEF-domain activity by binding a ligand or an interaction partner. How CdgC influences BldD activity remains to be uncovered in future work.

As in *S. coelicolor*, deletion of the PDEs *rmdA* or *rmdB* has an inhibitory effect on development in *S. venezuelae*, suggesting that in these strains BldD remains active as a repressor for a prolonged period, very likely due to elevated c-di-GMP levels. In *S. coelicolor*, both genes, *rmdA* and *rmdB*, must be deleted to achieve a block in differentiation and single deletions mildly inhibit spore production (Hull et al., 2012). In *S. venezuelae*, single deletions substantially delay the initiation of aerial mycelium formation and sporulation but after incubation of 4–5 days these strains develop mature green colonies with apparent wildtype morphology (Figure 2). As judged by *rmdA* and *rmdB* mutant phenotypes in these two *Streptomyces* species, it can be concluded that RmdA and RmdB are not redundant but impact a common signaling pathway. They also show a comparable expression pattern and belong to the most highly transcribed c-di-GMP metabolism genes (Figure 1A). However, RmdB is membrane-bound whereas RmdA is a cytosolic protein that may respond to specific signals perceived by the PAS–PAC sensory domain (Table 1 and Supplementary Figure S2). Moreover, deletion of *rmdB* arrests *S. venezuelae* in the vegetative growth phase for one day longer than deletion of *rmdA* (Figure 2), indicating that each of the two proteins also has a unique input into the development control.

CdgA, CdgB, CdgE, CdgC, RmdA, and RmdB are present in at least 90% of the *Streptomyces* strains, we have analyzed bioinformatically and thus represent the most important components of *Streptomyces* c-di-GMP-metabolism. Effects of *cdgA* deletion on development has not been addressed in any previous studies. But, in *S. coelicolor*, it has been shown that overexpression of CdgA with an intact GGDEF motif blocks the formation of aerial hyphae and the synthesis of the blue antibiotic actinorhodin, whereas overexpression of a mutagenized variant containing AADEF amino acids in the active site does not (den Hengst et al., 2010). Depending on the growth medium, overproduction of *cdgB* showed similar effects i.e., inhibition of aerial mycelium formation and actinorhodin synthesis in *S. coelicolor*. However, deletion of *cdgB* does not affect development of *S. coelicolor* when grown on SFM (soy flour mannitol) medium but delays development on R2 and R5 media. As uncovered in this work, CdgC is the key c-di-GMP producing enzyme with impact on developmental program progression since the *S. venezuelae cdgC* mutant has a pronounced developmental phenotype and bypasses aerial mycelium formation (Figure 2).

According to our western blot analysis, CdgE, CdgA, CdgF, HdgA, and HdgB are all expressed in wildtype (Figure 1B) but are not involved in developmental control, as judged by the mutant phenotypes (Supplementary Figure S3). It is likely that activating signals for these proteins are missing under the conditions tested. CdgD is the least conserved c-di-GMP turnover protein in *Streptomyces*, does not have any impact on development in *S. venezuelae* and was not detectable in our Western blot analysis (Figures 1B, 4 and Supplementary Figure S3). We cannot exclude that a relevant regulatory region is missing in our *cdgD*-FLAG construct, but in line with poor conservation of CdgD, it is likely that this GGDEF-protein is not produced in *S. venezuelae* and, e.g., post-transcriptional degradation could be involved.

The pan genome of 93 *Streptomyces* strains includes 15 genes encoding GGDEF/EAL/HD-GYP domains with the minimal set being five such genes, as found in, e.g., *Streptomyces sp.* CLI2509, and the maximal set being 12 genes, as found in e.g., *S. lincolnensis* NRRL2936. Four of the genes coding for c-di-GMP turnover enzymes found in the pan genome are unique to certain species or are present in very few species. Such diversity in the c-di-GMP metabolism genes in a genus is not unusual since e.g., in the genomes of *Azospirillum* strains, the number of genes encoding GGDEF/EAL proteins varies between 29 and 41 (Mata et al., 2018). However, the biological reasons underlying such diversification are difficult to explore but may involve adaptation to specific environmental niches.

## Materials and Methods

### Bacterial Strains, Growth Conditions, and Conjugations

*Escherichia coli* BW25113 (Datsenko and Wanner, 2000) containing a λ RED plasmid, pIJ790, was grown in LB under aeration at 30°C and used to create disruption cosmids for the generation of the DGC/PDE mutant strains and to replace the kan^R^ marker on the PI1_C11 cosmid with the *apr-oriT* region amplified from the pIJ773 template plasmid (Supplementary Table S1). HME68 (Thomason et al., 2014) was used for λ RED-mediated recombineering of the PI1_C11 *apr-oriT* cosmid with the single-strand oligonucleotide ssOligo ssRec5187 ALLEF (Supplementary Table S1) to create the G658A/G659L/D660L mutations in the *cdgC* locus on the cosmid. *E. coli* ET12567 containing pUZ8002 (Paget et al., 1999) was grown at 37°C and was used for conjugation experiments. *S. venezuelae* strains (Supplementary Table S1) were grown at 30°C in maltose-yeast extract-malt extract (MYM) medium (Stuttard, 1982) containing 50% tap water and 200 μl trace element solution (Kieser et al., 2000) per 100 ml and liquid cultures were inoculated with 10^6^ CFU/ml spores. Conjugations between *E. coli* and *S. venezuelae* were carried out as previously described (Bibb et al., 2012). For *S. venezuelae* macrocolonies or patches 12 μl spores (2 × 10^5^ CFU) in 20% glycerol were spotted on MYM-TAP agar and incubated at 30°C for up to 7 days.

### Generation of *S. venezuelae cdgC* G658A/G659L/D660L Mutant Strain

To create the *cdgC* G658A/G659L/D660L mutation on the PI1_C11 *kan*::*apr-oriT* cosmid, recombineering using single-strand oligonucleotide ssOligo ssRec5187 ALLEF (Supplementary Table S1), and *E. coli* HME68 strain was performed as described in Costantino and Court (2003). The mutagenized cosmid was confirmed by PCR and Sanger sequencing and transformed into *E. coli* ET12567/pUZ8002 for conjugation into *S. venezuelae*. Conjugation plates were incubated at room temperature overnight before overlaying with apramycin. Ex-conjugants were re-streaked once on plates containing apramycin and nalidixic acid and then several times on non-selective medium. The desired mutants arising from a double crossing over were screened for apramycin sensitivity followed by PCR to confirm the *cdgC* G658A/G659L/D660L mutation. A PCR product comprising the *cdgC* PAS-PAC-GGDEF region was sequenced and the resulting strain was named SVSN1.

### Construction of Complemented *S. venezuelae* Strains

For complementation analysis, *cdgC* and its promoter region were amplified using primers 5187-HindIII-pMS82-f and 5187-KpnI-pMS82-rev listed in Supplementary Table S1 and cloned into HindIII-KpnI cut pMS82 to create pSVOJ1. The plasmid was introduced into *S. venezuelae cdgC* G658A/G659L/D660L and Δ*cdgC* by conjugation, and the strains were named SVSN55 and SVSN3, respectively. The strain SVABK-4 resulted from the conjugation of pIJ10350 containing *cdgB* from *S. coelicolor* on the pIJ10257 vector into *S. venezuelae* Δ*cdgC*. To engineer *S. venezuelae* strains expressing c-di-GMP-proteins with a C-terminal, triple FLAG (MDYKDHDGDYKDHDIDYKDDDDK) affinity tag, the FLAG tag sequence was cloned into the pMS82 derivative pIJ10770 cut with XhoI and KpnI by using the 3xFLAG_XhoI_f and 3xFLAG_KpnI_r primers, creating p3xFLAG. The genes of interest including approximate 300 bp upstream of the open reading frames were then amplified using primers listed in Supplementary Table S1 and cloned in frame with the FLAG tag into the p3xFLAG vector. In the case of *cdgD*, *rmdA* and *hdgB* the corresponding upstream genes and their 5′ UTR were included in the constructs, due to potential operon organization. The resulting plasmids listed in Supplementary Table S1 were conjugated into the adequate c-di-GMP mutants, and the strains were subsequently used for Western blot analysis.

### Western Blotting

For FLAG-tag-based Western blot analysis, samples were taken after 10 (or 12 in the case of CdgE-FLAG and CdgF-FLAG), 14, 16, 18, and 20 h of growth. *S. venezuelae* wildtype served as negative control and was harvested after 20 h of incubation. After washing in 1× PBS, the cell pellets were resuspended in 1× PBS containing cOmplete Protease Inhibitor Cocktail Tablets (Roche) and lysed using a BeadBeater (Biozym; four cycles at 6.00 m/s; 30 s pulse; 60 s interval). Lysates were then centrifuged at 4500 rpm for 10 min at 4°C to remove cell debris and total protein concentration was determined using Bradford assay (Roth). Following amounts of total protein were loaded and separated on a SDS polyacrylamide gel: 5 μg for CdgB-FLAG; 10 μg for CdgE-FLAG, RmdB-FLAG and HdgB-FLAG; 15 μg for CdgA-FLAG, CdgC-FLAG, and RmdA-FLAG; 20 μg for CdgD-FLAG, CdgF-FLAG, HdgA-FLAG, and wildtype. Loading controls are shown in Supplementary Figure S1. After electrophoresis, proteins were transferred to a polyvinylidene difluoride (PVDF, Roth) membrane by electroblotting. For detection, anti-FLAG antibody (Sigma) was used. Bound primary antibody was visualized by using anti-mouse IgG-HRP (Thermo Fisher Scientific) secondary antibody and ECL chemiluminescent detection reagent (Perkin Elmer).

### Purification of CdgC and DGC Assay

For protein purification, the cytosolic part of *cdgC* was cloned into the pET15b (Novagen) vector using primers listed in Supplementary Table S1, which results in N-terminally His6-tagged gene product. *E. coli* Rosetta (Novagen) containing pET15b-ΔTM-*cdgC* (pSVSN2) was grown in LB with 100 μg/ml ampicillin at 37°C to an OD_578nm_ of 0.6. Protein expression was induced by adding IPTG (50 μM final concentration) and cultivation was continued overnight at 16°C. Cells were harvested and lysed by passage through a French press, and the soluble fraction was used for native protein purification using Ni-NTA affinity chromatography according to standard protocols (Qiagen). Buffer containing cOmplete Protease Inhibitor Cocktail Tablets (Roche) and 50 mM Tris, pH 8.0; 500 mM NaCl; 20 mM imidazole; 1 mM MgCl_2_; 5 mM β-mercaptoethanol; 0.1% Triton X 100 as well as 5% glycerol was used for lysis and washing steps. Prior activity assays, the purified protein was dialysed against a modified cyclase reaction buffer (25 mM Tris-HCl pH 7.5, 100 mM NaCl, 10 mM MnCl_2_, 5 mM β-mercaptoethanol, 5% glycerol).

Diguanylate cyclase reactions were performed according to Christen et al. (2005) with slight modifications. Two μM of purified His6-tagged ΔTM-CdgC in cyclase reaction buffer (see above) were incubated with 4 nM [α-^32^P]-GTP (Hartmann Analytic GmbH) at 30°C for 60 min. The reaction was stopped by heating to 95°C for 5 min and by mixing of 5 μl reaction with equal volume of 0.5 M EDTA pH 8.0. A reaction with purified PleD^∗^, a constitutively active DGC (Paul et al., 2007) was treated identically and served as positive control. Samples were separated on Polygram CEL 300 PEI cellulose TLC plates (Macherey–Nagel). Plates were developed in 1:1.5 (v/v) saturated NH_4_SO_4_ and 1.5 M KH_2_PO_4_, pH 3.6, and exposed on a Phosphor Imaging Screen (Fuji) after drying.

### Phase-Contrast Microscopy

Coverslips were placed on the surface of *S. venezuelae* colony and put on a thin agarose pad on a microscope slide for microscopy. Cells were imaged using the Leica DM2000 LED microscope at 100× magnification. Digital images were organized using ADOBE photoshop software.

### Pan Genome, Transcriptomic, and Domain Organization Analysis

*Streptomyces* species that had a complete genome assembly were downloaded from NCBI. A total of 93 genomes (Supplementary Table S2) were analyzed after excluding redundant genomes. Plasmids were excluded from the analysis and plasmid genbank records were removed from genbank files prior to conservation analysis. We employed the BPGA analysis tool (Chaudhari et al., 2016) for conservation and functional annotation of genes encoding c-di-GMP turnover enzymes. We set the id cutoff at 0.45. Downstream analyses were carried out using in-house python and perl scripts. Clusters containing c-di-GMP related genes were determined according to their COG annotations. The COG references used included: COG2199, COG2200, COG3706, COG3887, COG5001, COG2206, COG3437, COG3434, and COG4943. In addition, BldD was also included as it is known to conduct regulatory activity, which is modulated by c-di-GMP (Tschowri et al., 2014). The c-di-GMP clusters were classified into four categories: “core” (conserved in all 93 species), “soft core” (conserved in 88–92 species), “accessory” (conserved in 2–87 species), and “unique” (exclusively present in one species). We found that BPGA had few inconsistencies when protein ids% were around 50 or below. For the c-di-GMP metabolisms genes, NCBI blastp was used to validate the BPGA pipeline output for proteins that had 50% id or below. The modified output is highlighted in red in Supplementary Table S3. We also found some errors in the genbank files, and we highlighted those in green in Supplementary Table S3. Protein domain organization analysis was performed using SMART^2^.

### Microarray Analysis

Microarray data for the wildtype strain were acquired from the ArrayExpress database, accession number E-MEXP-3612 (Bibb et al., 2012). The raw data corresponding to each of the time course experiments were quantile normalized and median polished using the RMA method (Irizarry et al., 2003). The mean normalized expression values were calculated from the biological replicates and reported as log2. The expression figure was compiled using an in-house python script.

## Author Contributions

MA-B performed the bioinformatical analysis, made the figures, and wrote the paper. JH and SN performed the experiments and made the figures. SL performed the experiments. NT designed the study, created strains, made the figures, and wrote the paper. All authors read the manuscript.

## Conflict of Interest Statement

The authors declare that the research was conducted in the absence of any commercial or financial relationships that could be construed as a potential conflict of interest.

## Footnotes

1 https://www.ncbi.nlm.nih.gov

2 http://smart.embl-heidelberg.de

## References

[B1] Bibb M. J. Domonkos A. Chandra G. Buttner M. J. (2012). Expression of the chaplin and rodlin hydrophobic sheath proteins in *Streptomyces venezuelae* is controlled by sigma(BldN) and a cognate anti-sigma factor, RsbN. *Mol. Microbiol.* 84 1033–1049. 10.1111/j.1365-2958.2012.08070.x 22582857

[B2] Bush M. J. Chandra G. Findlay K. C. Buttner M. J. (2017). Multi-layered inhibition of *Streptomyces* development: BldO is a dedicated repressor of *whiB*. *Mol. Microbiol.* 104 700–711. 10.1111/mmi.13663 28271577 PMC5485038

[B3] Bush M. J. Tschowri N. Schlimpert S. Flardh K. Buttner M. J. (2015). c-di-GMP signalling and the regulation of developmental transitions in streptomycetes. *Nat. Rev. Microbiol.* 13 749–760. 10.1038/nrmicro3546 26499894

[B4] Chan C. Paul R. Samoray D. Amiot N. C. Giese B. Jenal U. (2004). Structural basis of activity and allosteric control of diguanylate cyclase. *Proc. Natl. Acad. Sci. U.S.A.* 101 17084–17089. 15569936 10.1073/pnas.0406134101 PMC535365

[B5] Chaudhari N. M. Gupta V. K. Dutta C. (2016). BPGA- an ultra-fast pan-genome analysis pipeline. *Sci. Rep.* 6:24373. 10.1038/srep24373 27071527 PMC4829868

[B6] Christen M. Christen B. Folcher M. Schauerte A. Jenal U. (2005). Identification and characterization of a cyclic di-GMP-specific phosphodiesterase and its allosteric control by GTP. *J. Biol. Chem.* 280 30829–30837. 15994307 10.1074/jbc.M504429200

[B7] Costantino N. Court D. L. (2003). Enhanced levels of lambda Red-mediated recombinants in mismatch repair mutants. *Proc. Natl. Acad. Sci. U.S.A.* 100 15748–15753. 10.1073/pnas.2434959100 14673109 PMC307639

[B8] Datsenko K. A. Wanner B. L. (2000). One-step inactivation of chromosomal genes in *Escherichia coli* K-12 using PCR products. *Proc. Natl. Acad. Sci. U.S.A.* 97 6640–6645. 10.1073/pnas.120163297 10829079 PMC18686

[B9] den Hengst C. D. Tran N. T. Bibb M. J. Chandra G. Leskiw B. K. Buttner M. J. (2010). Genes essential for morphological development and antibiotic production in *Streptomyces coelicolor* are targets of BldD during vegetative growth. *Mol. Microbiol.* 78 361–379. 20979333 10.1111/j.1365-2958.2010.07338.x

[B10] Flärdh K. Buttner M. J. (2009). *Streptomyces* morphogenetics: dissecting differentiation in a filamentous bacterium. *Nat. Rev. Microbiol.* 7 36–49. 10.1038/nrmicro1968 19079351

[B11] Hull T. D. Ryu M. H. Sullivan M. J. Johnson R. C. Klena N. T. Geiger R. M. (2012). Cyclic Di-GMP phosphodiesterases RmdA and RmdB are involved in regulating colony morphology and development in *Streptomyces coelicolor*. *J. Bacteriol.* 194 4642–4651. 10.1128/JB.00157-12 22753061 PMC3415515

[B12] Irizarry R. A. Hobbs B. Collin F. Beazer-Barclay Y. D. Antonellis K. J. Scherf U. (2003). Exploration, normalization, and summaries of high density oligonucleotide array probe level data. *Biostatistics* 4 249–264. 10.1093/biostatistics/4.2.249 12925520

[B13] Jones S. E. Ho L. Rees C. A. Hill J. E. Nodwell J. R. Elliot M. A. (2017). *Streptomyces* exploration is triggered by fungal interactions and volatile signals. *eLife* 6:e21738. 10.7554/eLife.21738 28044982 PMC5207766

[B14] Kieser T. Bibb M. J. Buttner M. J. Chater K. F. Hopwood D. A. (2000). *Practical Streptomyces Genetics.* Norwich: The John Innes Foundation.

[B15] Lindenberg S. Klauck G. Pesavento C. Klauck E. Hengge R. (2013). The EAL domain protein YciR acts as a trigger enzyme in a c-di-GMP signalling cascade in *E. coli* biofilm control. *EMBO J.* 32 2001–2014. 10.1038/emboj.2013.120 23708798 PMC3715855

[B16] Mata A. R. Pacheco C. M. Cruz Perez J. F. Saenz M. M. Baca B. E. (2018). In silico comparative analysis of GGDEF and EAL domain signaling proteins from the *Azospirillum* genomes. *BMC Microbiol.* 18:20. 10.1186/s12866-018-1157-0 29523074 PMC5845226

[B17] McCormick J. R. Flärdh K. (2012). Signals and regulators that govern *Streptomyces* development. *FEMS Microbiol. Rev.* 36 206–231. 10.1111/j.1574-6976.2011.00317.x 22092088 PMC3285474

[B18] Paget M. S. Chamberlin L. Atrih A. Foster S. J. Buttner M. J. (1999). Evidence that the extracytoplasmic function sigma factor sigmaE is required for normal cell wall structure in *Streptomyces coelicolor* A3(2). *J. Bacteriol.* 181 204–211. 9864331 10.1128/jb.181.1.204-211.1999 PMC103550

[B19] Paul R. Abel S. Wassmann P. Beck A. Heerklotz H. Jenal U. (2007). Activation of the diguanylate cyclase PleD by phosphorylation-mediated dimerization. *J. Biol. Chem.* 282 29170–29177. 17640875 10.1074/jbc.M704702200

[B20] Rigali S. Anderssen S. Naome A. van Wezel G. P. (2018). Cracking the regulatory code of biosynthetic gene clusters as a strategy for natural product discovery. *Biochem. Pharmacol.* 153 24–34. 10.1016/j.bcp.2018.01.007 29309762

[B21] Römling U. Galperin M. Y. Gomelsky M. (2013). Cyclic di-GMP: the first 25 years of a universal bacterial second messenger. *Microbiol. Mol. Biol. Rev.* 77 1–52. 10.1128/MMBR.00043-12 23471616 PMC3591986

[B22] Ryan R. P. Fouhy Y. Lucey J. F. Crossman L. C. Spiro S. He Y. W. (2006). Cell-cell signaling in *Xanthomonas campestris* involves an HD-GYP domain protein that functions in cyclic di-GMP turnover. *Proc. Natl. Acad. Sci. U.S.A.* 103 6712–6717. 16611728 10.1073/pnas.0600345103 PMC1458946

[B23] Sarenko O. Klauck G. Wilke F. M. Pfiffer V. Richter A. M. Herbst S. (2017). More than enzymes that make or break cyclic di-GMP-local signaling in the interactome of GGDEF/EAL domain proteins of *Escherichia coli*. *mBio* 8:e01639-17. 10.1128/mBio.01639-17 29018125 PMC5635695

[B24] Schlimpert S. Wasserstrom S. Chandra G. Bibb M. J. Findlay K. C. Flardh K. (2017). Two dynamin-like proteins stabilize FtsZ rings during *Streptomyces* sporulation. *Proc. Natl. Acad. Sci. U.S.A.* 114 E6176–E6183. 10.1073/pnas.1704612114 28687675 PMC5544309

[B25] Schmidt A. J. Ryjenkov D. A. Gomelsky M. (2005). The ubiquitous protein domain EAL is a cyclic diguanylate-specific phosphodiesterase: enzymatically active and inactive EAL domains. *J. Bacteriol.* 187 4774–4781. 15995192 10.1128/JB.187.14.4774-4781.2005 PMC1169503

[B26] Schumacher M. A. Zeng W. Findlay K. C. Buttner M. J. Brennan R. G. Tschowri N. (2017). The *Streptomyces* master regulator BldD binds c-di-GMP sequentially to create a functional BldD2-(c-di-GMP)4 complex. *Nucleic Acids Res.* 45 6923–6933. 10.1093/nar/gkx287 28449057 PMC5499655

[B27] Stuttard C. (1982). Temperate phages of *Streptomyces venezuelae*: lysogeny and host specificity shown by phages SV1 and SV2. *Microbiology* 128 115–121.

[B28] Tarutina M. Ryjenkov D. A. Gomelsky M. (2006). An unorthodox bacteriophytochrome from *Rhodobacter sphaeroides* involved in turnover of the second messenger c-di-GMP. *J. Biol. Chem.* 281 34751–34758. 16968704 10.1074/jbc.M604819200

[B29] Thomason L. C. Sawitzke J. A. Li X. Costantino N. Court D. L. (2014). Recombineering: genetic engineering in bacteria using homologous recombination. *Curr. Protoc. Mol. Biol.* 106 1 11–39. 10.1002/0471142727.mb0116s106 24733238

[B30] Tran N. T. Den Hengst C. D. Gomez-Escribano J. P. Buttner M. J. (2011). Identification and characterization of CdgB, a diguanylate cyclase involved in developmental processes in *Streptomyces coelicolor*. *J. Bacteriol.* 193 3100–3108. 10.1128/JB.01460-10 21515767 PMC3133206

[B31] Tschowri N. (2016). Cyclic dinucleotide-controlled regulatory pathways in *Streptomyces* species. *J. Bacteriol.* 198 47–54. 10.1128/JB.00423-15 26216850 PMC4686203

[B32] Tschowri N. Schumacher M. A. Schlimpert S. Chinnam N. B. Findlay K. C. Brennan R. G. (2014). Tetrameric c-di-GMP mediates effective transcription factor dimerization to control *Streptomyces* development. *Cell* 158 1136–1147. 10.1016/j.cell.2014.07.022 25171413 PMC4151990

[B33] Ventura M. Canchaya C. Tauch A. Chandra G. Fitzgerald G. F. Chater K. F. (2007). Genomics of Actinobacteria: tracing the evolutionary history of an ancient phylum. *Microbiol. Mol. Biol. Rev.* 71 495–548. 10.1128/MMBR.00005-07 17804669 PMC2168647

