# Supplementary material for: Expression Patterns, Genomic Conservation and Input Into Developmental Regulation of the GGDEF/EAL/HD-GYP Domain Proteins in Streptomyces
Source: Front Microbiol. 2018 Oct 23;9:2524. doi: 10.3389/fmicb.2018.02524 (PMC6205966; doi:10.3389/fmicb.2018.02524)
Supplement: Supplementary file 1 [file Table_1.docx]

| **Table S1. Strains, plasmids, and oligonucleotides used in this study.** | |  |  |
| --- | --- | --- | --- |
|  | Relevant genotype/comments | Source or reference |  |
| **Strains** |  |  |  |
| ***S. venezuelae*** |  |  |  |
| NRRL B-65442 | Wild type | (NCBI Reference Sequence: NZ_CP018074.1) |  |
| SVNT20 | *cdgC::apr*; Apr^R^ | This study | |
| SVNT21 | *cdgD::apr*; Apr^R^ | This study | |
| SVNT22 | *cdgE::apr*; Apr^R^ | This study | |
| SVNT23 | *cdgB::apr*; Apr^R^ | This study | |
| SVNT24 | *cdgA::apr*; Apr^R^ | This study | |
| SVNT25 | *cdgF::apr*; Apr^R^ | This study | |
| SVNT26 | *rmdB::apr*; Apr^R^ | This study | |
| SVNT27 | *rmdA::apr*; Apr^R^ | This study | |
| SVNT28 | *hdgA::apr*; Apr^R^ | This study | |
| SVNT29 | *hdgB::apr*; Apr^R^ | This study | |
| SVSN1 | *cdgC* (G658A/G659L/D660L) | This study | |
| SVSN3 | *cdgC::apr* *attB*_ΦBT1_::pMS82-*cdgC*; Apr^R^, Hyg^R^ | This study | |
| SVSN55 | *cdgC* (G658A/G659L/D660L) *attB*_ΦBT1_::pMS82-*cdgC*; Apr^R^, Hyg^R^ | This study | |
| SVSN56 | *cdgC* (G658A/G659L/D660L) *attB*_ΦBT1_::pMS82; Apr^R^, Hyg^R^ | This study | |
| SVSN57 | *cdgC::apr* *attB*_ΦBT1_::pMS82; Apr^R^, Hyg^R^ | This study | |
| SVABK-4 | *cdgC::apr*  *attB*_ΦBT1_::pIJ10257-*cdgB*; Apr^R^, Hyg^R^ | This study | |
| SVNT30 | *cdgA::apr; attB*_ΦBT1_*::*p3xFLAG*-cdgA*; Apr^R^, Hyg^R^ | This study | |
| SVNT31 | *rmdA::apr; attB*_ΦBT1_*::*p3xFLAG*-rmdA*; Apr^R^, Hyg^R^ | This study | |
| SVNT34 | *hdgA::apr; attB*_ΦBT1_*::*p3xFLAG*-hdgA*; Apr^R^, Hyg^R^ | This study | |
| SVNT35 | *hdgB::apr; attB*_ΦBT1_*::*p3xFLAG*-hdgB*; Apr^R^, Hyg^R^ | This study | |
| SVNT36 | *cdgF::apr; attB*_ΦBT1_*::*p3xFLAG*-cdgF*; Apr^R^, Hyg^R^ | This study | |
| SVNT37 | *cdgE::apr; attB*_ΦBT1_*::*p3xFLAG*-cdgE*; Apr^R^, Hyg^R^ | This study | |
| SVNT38 | *cdgD::apr; attB*_ΦBT1_*::*p3xFLAG*-cdgD*; Apr^R^, Hyg^R^ | This study | |
| SVNT39 | *rmdB::apr; attB*_ΦBT1_*::*p3xFLAG*-rmdB*; Apr^R^, Hyg^R^ | This study | |
| SVSL1 | *cdgB::apr; attB*_ΦBT1_*::*p3xFLAG*-cdgB*; Apr^R^, Hyg^R^ | This study | |
| SVSN6 | *cdgC::apr; attB*_ΦBT1_*::*p3xFLAG*-cdgC*; Apr^R^, Hyg^R^ | This study | |
| ***E. coli*** |  |  | |
| ET12567/pUZ8002 | *dam*, *dcm*, *hsd*, Kan^R^, Cm^R^ | (Paget et al., 1999) | |
| BW25113/pIJ790 | (Δ(*araD-araB*)*567*, Δ*lacZ4787*(::*rrnB-4*), *lacIp-4000*(lacI^Q^), λ-, *rpoS369*(Am), *rph-1*, Δ(*rhaD-rhaB*)568, *hsdR514*; Cm^R^ | (Datsenko and Wanner, 2000) | |
| HME68 | W3110 *galKtyr145UAG* Δ*lacU169* [λ *c*I*857* Δ*(crobioA)*]  *mutS*<>*cat;* defective methyl-directed mismatch repair system | (Thomason et al., 2014) | |
| Rosetta (DE3) | contains pRARE to supply tRNAs for rare codons; Cm^R^ | (Novagen) | |
| **Plasmids** |  |  | |
| pMS82 | Vector for conjugal transfer of DNA from *E. coli* to *Streptomyces*; integrates site-specifically at the ΦBT1 attachment site; Hyg^R^ | (Gregory et al., 2003) | |
| pIJ773 | Plasmid template for amplification of the *apr-oriT* cassette for ‘Redirect’ PCR-targeting | (Gust et al., 2003) | |
| pIJ790 | Modified l RED recombination plasmid [*oriR101*] [*repA101*(ts)] *araBp-gam-be-exo*, Cm^R^ | (Gust et al., 2003) | |
| pUZ8002 | RP4 derivative with defective oriT, Kan^R^ | (Paget et al., 1999) | |
| pIJ10257 | Plasmid integrating at the ϕBT1 *attB* attachment site containing the constitutive *ermEp** promoter, Hyg^R^ | (Hong et al., 2005) | |
| pIJ10350 | pIJ10257 carrying *cdgB* from *S. coelicolor* | (Tran et al., 2011) | |
| pSVOJ1 | pMS82-*cdgC* | This study | |
| pIJ10770 | pMS82 with an extended Multiple Cloning Site (MCS) lacking an intrinsic apramycin promoter upstream of the extended MCS | (Schlimpert et al., 2017) | |
| p3xFLAG | pIJ10770 containing triple Flag Tag downstream of the MCS in the XhoI/KpnI sites | This study | |
| pSVSL-4 | pSS170 *cdgA*-FLAG | This study | |
| pSVSL-11 | pSS170 *cdgB*-FLAG | This study | |
| pSVSN-23 | pSS170 *cdgC*-FLAG | This study | |
| pSVSL-3 | pSS170 *cdgD*-FLAG | This study | |
| pSVSL-5 | pSS170 *cdgE*-FLAG | This study | |
| pSVSL-6 | pSS170 *cdgF*-FLAG | This study | |
| pSVJH01 | pSS170 *rmdA*-FLAG | This study | |
| pSVJH02 | pSS170 *rmdB*-FLAG | This study | |
| pSVNT-11 | pSS170 *hdgA*-FLAG | This study | |
| pSVNT-12 | pSS170 *hdgB*-FLAG | This study | |
| pSVSN-2 | pET15b-∆TM-*cdgC* | This study | |

| **Oligonucleotides used for amplification of the *oriT-apr* cassette with gene-specific extensions** | |
| --- | --- |
| Oligonucleotide | 5′-3′ Sequence |
| *cdgC*_H1-P1-fw | GCGGCCGGGTGCGGTGGCGCGGCTCCGGAGGGACCGGTGATTCCGGGGATCCGTCGACC |
| *cdgC*_H2-P2-rev | CATGGCACTGACCCTCCCTCTCCGCCCTCACCACTGTCATGTAGGCTGGAGCTGCTTC |
| *cdgD*_H1-P1-fw | CGTCGTCCGCGACATGCAGAAGGGCCGGTAGCGGCCATGATTCCGGGGATCCGTCGACC |
| *cdgD*_H2-P2-rev | CGGAAGTTTGGCGACCGAGGCCGCTCCGGGGGCCGCTCATGTAGGCTGGAGCTGCTTC |
| *cdgE*_H1-P1-fw | AAGGGAAAACGGATCAAACCTCCCTAAGCTGGAGGAATGATTCCGGGGATCCGTCGACC |
| *cdgE*_H2-P2-rev | ACGCCCCCCCGAGGGGAAGCCCGCGCCCGCGGCTCCTCATGTAGGCTGGAGCTGCTTC |
| *cdgB*_H1-P1-fw | CTGGAGTTGCTGTCGAGGTCTCGGGGAGAGCGAGCGATGATTCCGGGGATCCGTCGACC |
| *cdgB*_H2-P2-rev | CGAGCCCCCGAAGCGGCCCCGGGCGTGTCAGGAGCCTCATGTAGGCTGGAGCTGCTTC |
| *cdgA*_H1-P1-fw | ACATGTGACCGGCCCGACTGCACTACGGGAGCGAGCGTGATTCCGGGGATCCGTCGACC |
| *cdgA*_H2-P2-rev | TGCCTACCGCTCAGACCCGTACCGGCTCCGTACCGTTCATGTAGGCTGGAGCTGCTTC |
| *cdgF*_H1-P1-fw | CCCGACTCCGTCGCCGAGGGGACCCGGCTCCTGCCCGTGATTCCGGGGATCCGTCGACC |
| *cdgF*_H2-P2-rev | GGGGCGGCGCCGGCGGGCGCGGCCCGGTCCCGTGCGTCATGTAGGCTGGAGCTGCTTC |
| *rmdB*_H1-P1-fw | ACACCGACGGCACCGCCGCCTGTGAGAGGGACGGAAATGATTCCGGGGATCCGTCGACC |
| *rmdB*_H2-P2-rev | GGTTTGCCCGGGGTCGTCGCGCCGGGGTGACACGGCCTATGTAGGCTGGAGCTGCTTC |
| *rmdA*_H1-P1-fw | CGCCTTCTCCGGTTTCGCAGGGGTGGGGCGCAAGGCGTGATTCCGGGGATCCGTCGACC |
| *rmdA*_H2-P2-rev | CTTCGCGCGGGGAGGCCGGTTTCGTGCGTCCGGGTCTCATGTAGGCTGGAGCTGCTTC |
| *hdgA*_H1-P1-fw | GCGGCACGCCCGCGACGGCCTCCCGCGTATATCAGCTTGATTCCGGGGATCCGTCGACC |
| *hdgA*_H2-P2-rev | TCATGCGCGTTCCCTGGGCCGGGGCGGCAGCGGCGCGCCTGTAGGCTGGAGCTGCTTC |
| *hdgB*_H1-P1-fw | CCCGGCGCCCTCACCGTCGGCGCCGTGCACGGGGCCGCCATTCCGGGGATCCGTCGACC |
| *hdgB*_H2-P2-rev | CCATGCCCGGTCGGACGCGCGCTCATTACCCATGGGTTATGTAGGCTGGAGCTGCTTC |
| **Oligonucleotides used for verification of deletion mutants** | |
| *cdgC*-HindIII-MS82-fw | GGTGGTAAGCTTGTCACGGAGAGTACTGCCAC |
| *cdgC*-KpnI-MS82-rev | AGTAGTGGTACCCATCTCTTCTCGAAGCAGAG |
| *cdgD*-test-fw | GACACCACGATCAACAAGTAG |
| *cdgD*-KpnI-MS82-rev | AGTAGTGGTACCGTAGCGAAGCCGTCGCAGTC |
| *cdgE*-test-fw | CCTTCAACGAGAAGCGGAAG |
| *cdgE*-test-rev | CAGCACCCTACGGATTGAAG |
| *cdgB*-HindIII-MS82-fw | GGTGGTAAGCTTCACACCCGCACGCTAGTC |
| *cdgB-*KpnI-MS82-rev | AGTAGTGGTACCGGTTCCTCTCCATGCTCGC |
| *cdgA*-HindIII-MS82-fw | GGTGGTAAGCTTCACGTGGACCTCCGGTTCG |
| *cdgA*-KpnI-MS82-rev | AGTAGTGGTACCCGCTGAGTCCCGCATGAAC |
| *cdgF*-test-fw | CTGCCACACCGACGAGCTGAC |
| *cdgF*-test-rev | GCATCAGCGGATAGAGGTTG |
| *rmdB*-HindIII-MS82-fw | GGTGGTAAGCTTGAAGCTCACCCGTTCGGCG |
| *rmdB*-KpnI-MS82-rev | AGTAGTGGTACCGGTGAGCTTGATGGTGTGG |
| *rmdA*-test-fw | TCGTCCTCACCCACGCCTC |
| *rmdA*-test-rev | TGAGTGTTACCGTCCTACCG |
| *hdgA*-test-fw | GCAGCGACGACGTCCCCAC |
| *hdgA*-test-rev | TTCGCCGACGGCGGGTGC |
| *hdgB*-test-fw | CTTCGACCCCCGGATGGT |
| *hdgB*-test-rev | TCCTCGTACCGTCCGTCC |
| **Oligonucleotides used for cloning of *cdgC* into pMS82 and for amplification of *cdgC* (G658A/G659L/D660L) PAS-PAC-GGDEF region for sequencing** | |
| 5187-HindIII-pMS82-f | CAGAAGCTTGAGCCTGTGAACGTCCTC |
| 5187-KpnI-pMS82-rev | CAGGGTACCGCATCTCTTCTCGAAGCAG |
| 5187-NdeI-pET15b-GGDEF-fw | CAGCATATGATCATGATCGCCGCTCCC |
| 5187_pET15b-XhoI-PPG-rev | GACTCTCGAGTTAGACCACCTCGGCCTG |
| **ssOligonucleotides used for generation of G658A/G659L/D660L mutations in *cdgC* on the PI1_C11 cosmid** | |
| ssRec5187 ALLEF | GGCCGGGGACACGGCCGCCCGGCTCGCGCTCCTCGAGTTCGCCGCCCTCATCCTGGGCGACGGCGGACGC |
| **Oligonucleotides used for specific amplification of *cdgC* (G658A/G659L/D660L) for selection of clones containing the desired mutation** | |
| 5187-NdeI-pET15b-GGDEF-fw | CAGCATATGATCATGATCGCCGCTCCC |
| test_5187-ALLEF-rev | CGGCGAACTCGTCCGCCG |
| **Oligonucleotides used for amplification of the *oriT-apr* cassette with *neo*-specific extensions for replacement of the kan^R^ cassette on the PI1_C11 cosmid and for verification of the exchange** | |
| neo_H1-P1-fw | AGATCTGATCAAGAGACAGGATGAGGATCGTTTCGCatgATTCCGGGGATCCGTCGACC |
| neo_H2-P2-rev | TCGCTTGGTCGGTCATTTCGAACCCCAGAGTCCCGCtcaTGTAGGCTGGAGCTGCTTC |
| neo_test-fw | GTTTTATGGACAGCAAGCG |
| neo_test-rev | GAATCGAAATCTCGTGATG |
| **Oligonucleotides used for generation of the Flag constructs and for overexpression of ∆TM-*cdgC*** | |
| 5058_NdeI-f | GCTGCATATGCTCCCGGCCCCCTCG |
| 5058_AvrII-r | CATGCCTAGGCTGGACGACCTGGCCG |
| 6683-4_HindIII-for | GATCAAGCTTCAACGCGCGGGATTCGC |
| 6684_XhoI-rev | GCAGCCTCGAGGACGGCGTCCGCCAG |
| sven15_3908_XhoI-r | GCAGCCTCGAGGTGTGTGTTGCGCCTGC |
| sven15_3910_NdeI-f | GCTGCATATGGAACCGCTTCCACCACGT |
| 4502_AvrII-rev | CATGCCTAGGCCCGCCCCCGTCCC |
| 4502_NdeI-for | GCTGCATATGGTGGAGGCGGCGGA |
| sven15_3942_NdeI-f | GCTGCATATGGGGCCCTCGGGGGG |
| sven15_3942_NruI-r | CATGTCGCGAGCCGGCCCGCCGG |
| 2547_AvrII-r | CATGCCTAGGGACCCGCAGCGGTTCC |
| 2547_NdeI-f | GCTGCATATGGGCGCGCGGGGTCGA |
| sven15_0422_AvrII_r | CATGCCTAGGCGGCTCCGGCGTCCG |
| sven15_0422_NdeI_f | GCTGCATATGGCCGAACCGAGCGGAC |
| 4769-70_NdeI-for | GCTGCATATGGACACGGACTGCACCAAG |
| 4769-FLAG_XhoI-rev | GCAGCCTCGAGTGCGCGTTCCCTGGGC |
| 4770-FLAG_XhoI-rev | GCAGCCTCGAGCCCAGCGGGGGCGGA |
| 5187-HindIII-pMS82-f | CAGAAGCTTGAGCCTGTGAACGTCCTC |
| sven15_5080_XhoI_r | GCAGCCTCGAGAGTGGGTGGGACAGGCG |
| 3xFLAG_XhoI_f | CATCTCTCGAGGACTACAAGGAC |
| 3xFLAG_KpnI_r | CACTGGTACCCTACTTGTCGTCATCGTC |
| 5187_pET15b-NdeI-PPGEoTM-fw | GACCATATGATCATGCTGCTCGACAAC |
| 5187-XhoI-pET15b-EAL-rev | CAGTCTCGAGTTCAAGTGGGTGGGACA |

**Supplementary References**

Datsenko, K.A., and Wanner, B.L. (2000). One-step inactivation of chromosomal genes in *Escherichia coli* K-12 using PCR products. *Proc Natl Acad Sci U S A* 97(12)**,** 6640-6645. doi: 10.1073/pnas.120163297.

Gregory, M.A., Till, R., and Smith, M.C. (2003). Integration site for *Streptomyces* phage phiBT1 and development of site-specific integrating vectors. *J Bacteriol* 185(17)**,** 5320-5323.

Gust, B., Challis, G.L., Fowler, K., Kieser, T., and Chater, K.F. (2003). PCR-targeted *Streptomyces* gene replacement identifies a protein domain needed for biosynthesis of the sesquiterpene soil odor geosmin. *Proc Natl Acad Sci U S A* 100(4)**,** 1541-1546. doi: 10.1073/pnas.0337542100.

Hong, H.J., Hutchings, M.I., Hill, L.M., and Buttner, M.J. (2005). The role of the novel Fem protein VanK in vancomycin resistance in *Streptomyces coelicolor*. *J Biol Chem* 280(13)**,** 13055-13061. doi: 10.1074/jbc.M413801200.

Paget, M.S., Chamberlin, L., Atrih, A., Foster, S.J., and Buttner, M.J. (1999). Evidence that the extracytoplasmic function sigma factor sigmaE is required for normal cell wall structure in *Streptomyces coelicolor* A3(2). *J Bacteriol* 181(1)**,** 204-211.

Schlimpert, S., Wasserstrom, S., Chandra, G., Bibb, M.J., Findlay, K.C., Flardh, K., et al. (2017). Two dynamin-like proteins stabilize FtsZ rings during *Streptomyces* sporulation. *Proc Natl Acad Sci U S A* 114(30)**,** E6176-E6183. doi: 10.1073/pnas.1704612114.

Thomason, L.C., Sawitzke, J.A., Li, X., Costantino, N., and Court, D.L. (2014). Recombineering: genetic engineering in bacteria using homologous recombination. *Curr Protoc Mol Biol* 106**,** 1 16 11-39. doi: 10.1002/0471142727.mb0116s106.

Tran, N.T., Den Hengst , C.D., Gomez-Escribano, J.P., and Buttner, M.J. (2011). Identification and characterization of CdgB, a diguanylate cyclase involved in developmental processes in *Streptomyces coelicolor*. *J Bacteriol.* 193(12)**,** 3100-3108.
