# Supplementary material for: Expression Patterns, Genomic Conservation and Input Into Developmental Regulation of the GGDEF/EAL/HD-GYP Domain Proteins in Streptomyces
Source: Front Microbiol. 2018 Oct 23;9:2524. doi: 10.3389/fmicb.2018.02524 (PMC6205966; doi:10.3389/fmicb.2018.02524)
Supplement: Supplementary file 4 [file Data_Sheet_1.docx]

Supplementary Material

**Expression patterns, genomic conservation and input into developmental regulation of the GGDEF/EAL/HD-GYP domain proteins in *Streptomyces***

**Mahmoud M. Al-Bassam, Julian Haist^†^, Sara Alina Neumann^†^, Sandra Lindenberg, and Natalia Tschowri***

*** Correspondence:** Corresponding author: [natalia.tschowri@hu-berlin.de](mailto:natalia.tschowri@hu-berlin.de)

**^†^** These authors contributed equally to this work


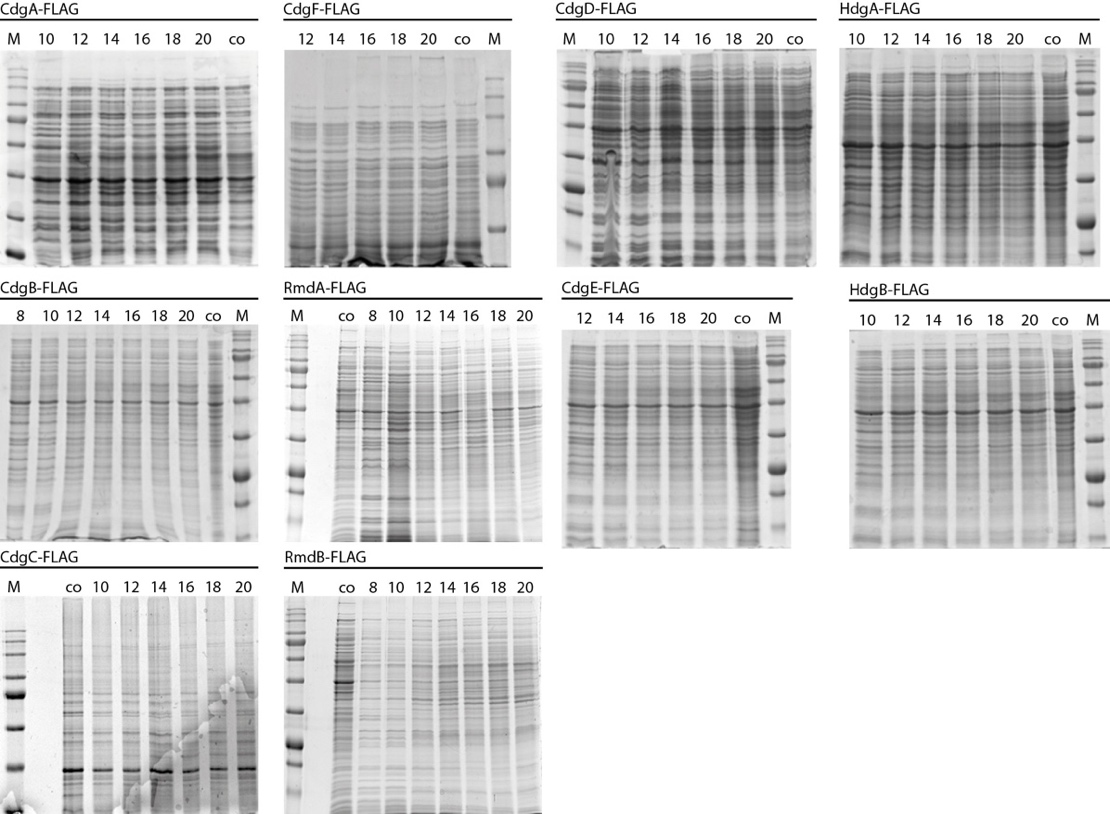


**Supplementary Figure 1. Loading controls for western blot analysis of expression patterns of FLAG-tagged c-di-GMP metabolizing proteins.** C-terminally FLAG-tagged alleles of genes coding for c-di-GMP metabolizing proteins were introduced using the integrative pIJ10770 vector into the corresponding mutants and expressed under the control of their native promoters from the ΦBT1 integration site. Strains were grown in liquid MYM at 30°C and samples were taken every 2 hours after indicated time of growth (numbers above the lanes). A *S. venezuelae* wildtype sample was harvested after 20 hours of growth and served as negative control (co). Whole cell lysates were prepared as described in M&M and the protein concentration was determined using the Bradford (Roth) assay. For each strain, same amounts of whole cell proteins were loaded on two SDS polyacrylamide gels that were run in parallel in SDS buffer at 50 mA for ca. 1 hour. One gel was used for western blotting and detection of FLAG-tagged protein using the anti-FLAG antibody (Fig. 1B). The other gel was stained with Coomassie and used as loading control. Following amounts of total protein were used: 5 µg for CdgB-FLAG; 10 µg for CdgE-, RmdB- and HdgB-FLAG; 15 µg for CdgA-, CdgC-, and RmdA-FLAG; 20 µg for CdgD-, CdgF-, HdgA-FLAG and wildtype.


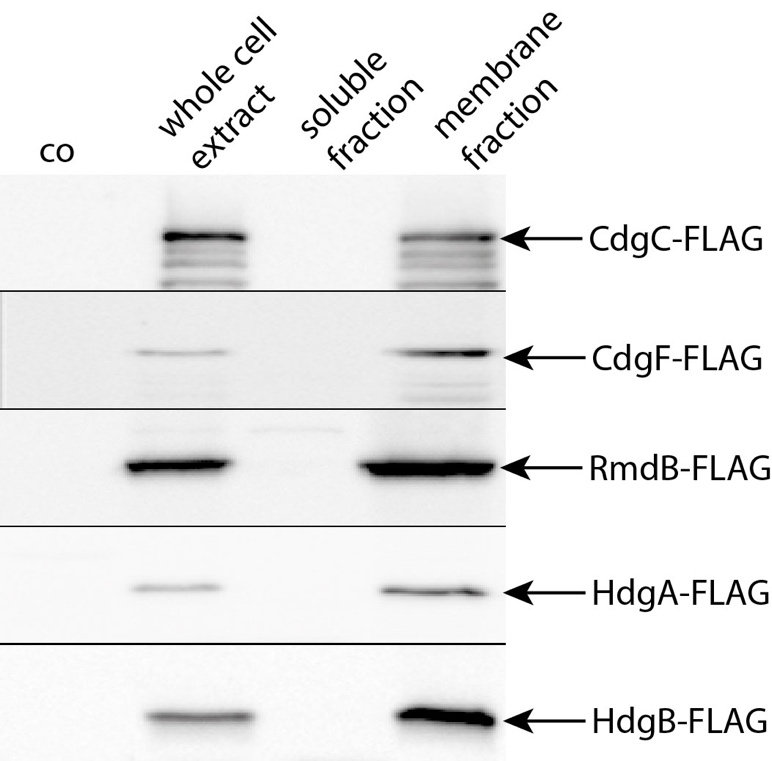


**Supplementary Figure 2: CdgC, CdgF, RmdB, HdgA and HdgB are membrane proteins.** To experimentally validate the predicted membrane-bound localization of CdgC, CdgF, RmdB, HdgA and HdgB (see Table 1), FLAG-tagged alleles were expressed from the ΦBT1 integration site in the relevant mutants. 5 ml of cells grown in liquid MYM for 20 hours at 30°C were harvested and centrifuged for 10 minutes at 5000 rpm. Cell pellets were resuspended in 1 x PBS containing cOmplete Protease Inhibitor Cocktail Tablets (Roche) and lysed using a BeadBeater. An aliquot of the whole cell fraction was saved for further analysis. The cytosolic fraction was separated from the membrane cell fraction using ultracentrifugation for 30 minutes at 66.000 rpm and the supernatant was saved. The pellet containing the cellular membrane material was washed 2 x with 1 x PBS with protease inhibitor and resuspended in 100 µl 1 x PBS containing cOmplete Protease Inhibitor Cocktail Tablets (Roche). Total protein concentration was determined using Bradford assay (Roth) and 20 µg total protein of each fraction were separated using SDS-PAGE. After electrophoresis, proteins were transferred to a polyvinylidene difluoride (PVDF, Roth) membrane by electroblotting. For detection, anti-FLAG antibody (Sigma) was used. Bound primary antibody was visualized by using anti-mouse IgG-HRP (Thermo Fisher Scientific) secondary antibody and ECL chemiluminescent detection reagent (Perkin Elmer). The membrane fraction from wildtype served as negative control (co).


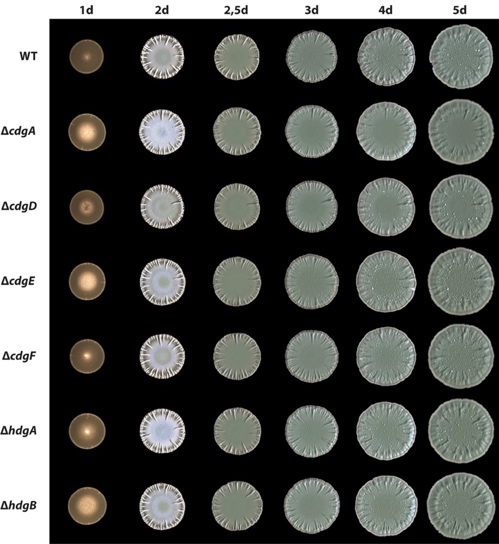


**Supplementary Figure 3.** **Deletion of *cdgA*, *cdgD*, *cdgE*, *cdgF*, *hdgA* and *hdgB* does not affect morphological differentiation in *S. venezuelae*.** Wildtype and mutant strains were grown on MYM agar for 5 days at 30°C. Images were taken after the indicated time of incubation.


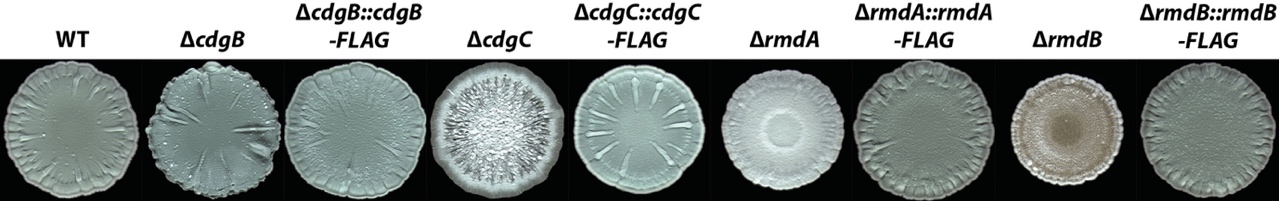


**Supplementary Figure 4. Expression of *cdgB*-FLAG, *cdgC*-FLAG, *rmdA*-FLAG and *rmdB*-FLAG complements the developmental mutant phenotype of the corresponding deletion strains.** FLAG-tagged alleles of *cdgB*, *cdgC*, *rmdA* and *rmdB* were introduced using the integrative pIJ10770 vector into the corresponding mutants and expressed under the control of their native promoters from the ΦBT1 integration site. Strains were grown on MYM agar for 4 days.

**Supplementary Figure 5. Analysis of gene-context conservation for *bldD*, *cdgB*, *cdgA*, *rmdA* and *rmdB*.** For synteny analysis, the three genes flanking upstream and downstream regions of *bldD*, *cdgB*, *cdgC*, *rmdB* and *rmdA*in each *Streptomyces* species (where an orthologue exists) were used to determine the conservation of the genetic context. Setting *S. venezuelae* as our reference, we considered the reciprocal top blast hit identity percent of each set of genes as a measure of the conservation of the flanking genes. The identity percent was plotted as heatmaps ranging from 0 to 1. The pipeline code was written in python and perl languages. The code is available upon request. The relevant c-di-GMP metabolic gene in each panel is shown in red font. The species carrying each orthologous gene is shown on the left.

|  |  |
| --- | --- |
